# Genetic and phenotypic heterogeneity of type 2 diabetes across Russian ancestry groups

**DOI:** 10.3389/fendo.2025.1672403

**Published:** 2025-09-10

**Authors:** Ekaterina E. Markelova, Irina V. Kononenko, Anatoliy Zubritskiy, Elizaveta Podshivalova, Alina Matrosova, Evgeniya Plaksina, Saleem Mansour, Pavel Ahtyamov, Elena Shagimardanova, Gulnar R. Vagapova, Nadezhda Maksimova, Liubov A. Sydykova, Diana S. Avzaletdinova, Tatyana V. Morugova, Petimat M. Dzhambetova, Marina V. Shestakova, Natalia G. Mokrysheva, Yulia A. Medvedeva

**Affiliations:** ^1^ Research Center of Biotechnology, Institute of Bioengineering, Russian Academy of Sciences, Moscow, Russia; ^2^ MSU Institute for Artificial Intelligence, Lomonosov Moscow State University, Moscow, Russia; ^3^ Endocrinology Research Center, Moscow, Russia; ^4^ Moscow Center for Advanced Studies, Moscow, Russia; ^5^ Skolkovo Institute of Science and Technology, Moscow, Russia; ^6^ Life Improvement by Future Technologies (LIFT) Center, Moscow, Russia; ^7^ Kazan State Medical Academy, Kazan, Russia; ^8^ North-Eastern Federal University, Yakutsk, Russia; ^9^ Bashkir State Medical University, Ufa, Russia; ^10^ Chechen State University named after A. A. Kadyrov, Grozny, Russia

**Keywords:** type 2 diabetes, genotyping, ancestry, risk scores, genetic mechanism, Russian population, genetic association, beta cell dysfunction

## Abstract

**Introduction:**

Type 2 diabetes mellitus (T2D) is a highly polygenic disease involving multiple biological pathways. Genetic ancestry may influence the predominant mechanisms driving T2D. Understanding how genetic background shapes T2D risk is crucial for developing personalized prevention and treatment strategies.

**Methods:**

We analyzed ancestry-specific differences in T2D mechanisms and assessed the prevalence of T2D-associated genetic clusters, reflecting biological mechanisms underlying T2D onset and progression, in individuals from three Russian ancestry groups: Chechens, Tatars, and Yakuts. Previously developed polygenic scores were applied to evaluate cluster prevalence and clinical risk factors across ancestry groups.

**Results:**

Cluster-specific polygenic scores varied significantly between populations. Yakuts exhibited higher scores for β-cell dysfunction, hyper-insulin secretion, and lipid metabolism alterations, whereas Chechens and Tatars had higher scores for obesity-related mechanisms.

**Discussion/Conclusions:**

The predominant mechanisms underlying T2D differ across populations. These ancestry-specific differences should be considered in public health recommendations and personalized medicine approaches.

## 1 Introduction

The multifactorial nature of type 2 diabetes mellitus (T2D) has been confirmed by numerous studies. The disease is influenced by multiple risk factors, including lifestyle, environment, and genetics (1, 2). The main pathophysiological processes underlying T2D development focus on the role of obesity, visceral and ectopic fat accumulation, insulin resistance, and β cell dysfunction (3).

The expanding catalog of genetic differences among humans is helping researchers understand why some individuals and populations are more prone to common diseases, such as T2D. Additionally, we are gaining insights that may improve the efficacy and safety of therapeutic drugs. Genome-wide association studies (GWAS) have identified over 600 genetic loci associated with T2D, many of which are involved in beta-cell function, insulin secretion, and insulin sensitivity (4). Genetic ancestry is one of the key factors determining predisposition to diabetes (5). The contribution of T2D-associated loci to disease risk varies significantly across populations due to differences in allele frequencies and population-specific genetic architecture (6). Rare variants tend to be specific to certain populations (7). Ancestry-specific differences in the balance between insulin secretion and insulin resistance further contribute to T2D heterogeneity (8). For instance, East Asian populations tend to have a higher prevalence of beta-cell dysfunction, while European populations often exhibit stronger associations with insulin resistance (8). These pathophysiological differences have a crucial impact on the appropriate preventive and therapeutic approaches, highlighting the need for population-specific research.

Russia is a multinational country with more than 190 ethnic groups, making it a unique setting for studying the genetic architecture of T2D in a diverse population (9). However, most existing population studies lack ancestral diversity and predominantly focus on Western European individuals (10). Recent studies have shown that allele frequencies of various clinically significant single nucleotide polymorphisms (SNPs) differ significantly between Russian and Western European populations (11). This suggests that unique population-specific variants may influence the genetic risk of T2D in Russia. Populations within Russia also exhibit differences in the prevalence of carbohydrate metabolism disorders and T2D (12). These findings highlight the importance of population-specific research in refining subtype classification and improving personalized treatment strategies.

Advances in phenotype-based clustering have revealed distinct subtypes of T2D, each with unique genetic, clinical, and pathophysiological characteristics (13). Recent studies by several groups of authors (14, 15) have leveraged genome-wide association study (GWAS) summary statistics to connect genetic loci to possible T2D pathological pathways by clustering genetic loci based on shared patterns of associations across multiple traits using a soft clustering approach. Smith et al. (14) have identified twelve genetic clusters and used them to generate partitioned polygenic scores (pPGSs) linked to distinct cellular and clinical features. In this work, we applied pPGSs to distinct populations in Russia to investigate the effect of ancestry on genetic predisposition to certain mechanisms, while keeping in mind that pPGSs reflect the likelihood of certain disease mechanisms occurring rather than a definitive indicator of individual risk of disease development.

## 2 Materials and methods

### 2.1 Study design and sample collection

**Figure 1:**
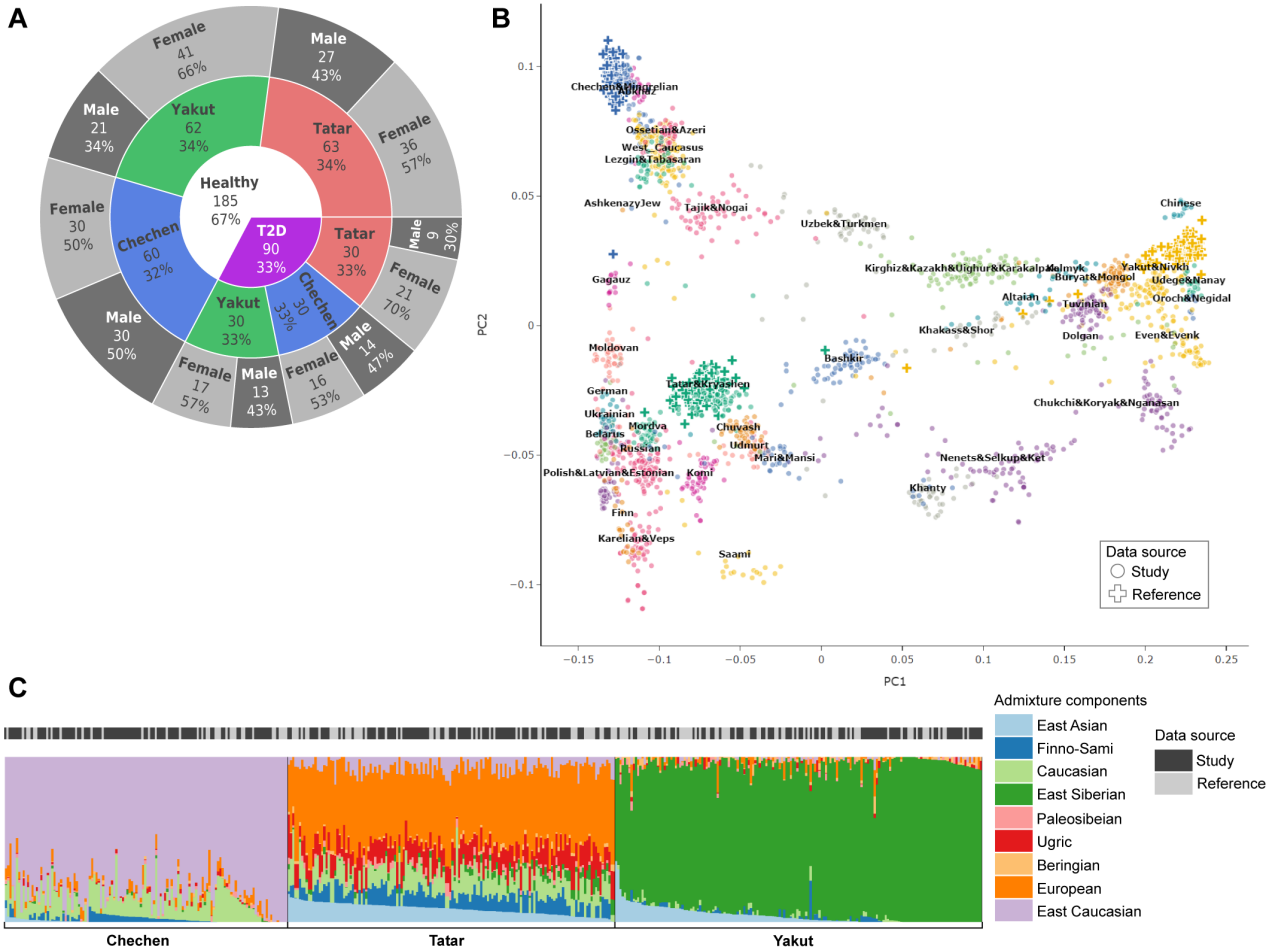
Exploratory analysis of the genetic ancestry in the study dataset. **(A)** Annotation structure of the study dataset. **(B)** PCA projection of study participants (N=275) and ancestry reference donors (N=1878). **(C)** ADMIXTURE component profiles for K=9. Study participants are marked in dark gray, and reference donors are marked in light gray.

The study included healthy individuals and patients with T2D from distinct ancestry groups: Yakuts (Yakutsk), Tatars (Kazan), and Chechens (Grozny), all of whom provided informed consent to participate. Ancestry was determined through self-reported data from participants and two generations of their ancestors. A total of 275 eligible participants were included in this observational study, which did not influence patient management. Patients with T2D were recruited at the primary healthcare level in three locations across Russia. Inclusion criteria were based on medical history, including a prior diagnosis of diabetes and the use of glucose-lowering drugs. T2D diagnosis followed WHO criteria (1999). Exclusion criteria included: 1) individuals with other types of diabetes; 2) close relatives of other study participants; and 3) pregnant or lactating individuals. Participants were divided into three groups based on self-reported ancestry: Yakuts (62 healthy individuals and 30 T2D patients), Tatars (63 healthy individuals and 30 T2D patients), and Chechens (60 healthy individuals and 30 T2D patients) (**Figure 1A**). All participants underwent the following assessments:

- Admission survey. Participants completed a survey detailing their supposed ancestry and that of their relatives across two generations.
- Anthropometric measurements. Height, weight, BMI, waist circumference (WC), and hip circumference (HC) were recorded. BMI was calculated as weight (kg)/(height(m))^2, and waist-hip ratio (WHR) was calculated as waist circumference (cm)/hip circumference (cm).
- Laboratory tests. Glycosylated hemoglobin (HbA1c) and blood lipids: total cholesterol (TC), LDL, HDL, and triglycerides (TG), were measured. Tests were carried out in local laboratories. The atherogenic index was calculated as (TC - HDL)/HDL, with all metrics in mmol/L.

### 2.2 Genotyping and data processing

Fasting morning venous blood samples were collected and stored at −80°C in a central biobank. Genomic DNA was isolated using the MagPure Universal DNA Kit (Magen Biotechnology, China) and was quantified and assessed using NanoDrop OneC (ThermoFS, USA). 200–500 ng of DNA samples were used for genotyping. Genotyping was performed using the 450K Infinium Global Screening Array (GSA) at the Endocrinology Research Center, Moscow. Data preprocessing and export to PACKEDPED format were conducted using Illumina GenomeStudio (16). The PLINK package (ver. 2.00a5LM) (17) was used to convert files to VCF (Variant Call Format), and the bcftools package (ver. 1.20) was employed to correct the reference allele based on the forward strand of the genomic reference hg38 (GRCh38.p14). This process led to the detection of 548,502 SNPs.

### 2.3 Ancestry reference data collection

For genetic ancestry analysis, an ancestry-specific reference dataset (N=1,878) was compiled from publicly available sources (18–29), capturing a range of ancestries present in Russia, though not all ancestries are fully represented (**Figure 1B**, **Supplementary Figure S1**). Due to the different genotyping platforms, only 40,566 SNPs were successfully detected in all individuals in the reference set.

### 2.4 Phasing and imputation

Study and reference data were phased with Eagle (ver. 2.4.1) and imputed using Beagle (ver. 5.4) with default parameters (burnin = 6, iterations = 12, imp-segment = 6, ne = 1000000). The 1000 Genomes Project (phase 3, N=3203) served as the reference for imputation. Variants with an imputation quality (DR2) ≥ 0.3 were retained, resulting in 10,314,190 SNPs for downstream analysis.

### 2.5 Determination of genetic ancestry

Global genetic ancestry was assessed using principal component analysis (PCA, hail package (30) and admixture proportion inference (ADMIXTURE) (31). ADMIXTURE was run with K ranging from 2 to 15, with K=9 selected based on cross-validation results. Admixture (ancestral) components were named based on their distribution in populations and following previous admixture studies of Russian samples (9, 18, 21). Two publicly available samples with ancestry profiles significantly deviating from their reported population averages were excluded (**Supplementary Methods**). All the 275 study participants’ inferred ancestries matched their self-reported data (**Figures 1A-C**).

### 2.6 Comparison of anthropometric and clinical measurements between populations

Linear and logistic regression analyses were conducted using the ordinary least squares (OLS) and Logit functions, respectively, from the statsmodels library (32). Non-normally distributed clinical measurements (e.g., BMI, TG, WC, HC, WHR, blood pressure, TC, HDL, atherogenic index, HbA1c) were log-transformed to achieve normal distribution, following established methodologies (33). The standardized beta coefficient refers to how many standard deviations the outcome variable will change per a standard deviation increase in the predictor variable. P-values for regression coefficients were obtained via the standard two-tailed t-tests and significance was assessed after correction for multiple comparisons using the Benjamini–Hochberg procedure.

### 2.7 Calculation of partitioned polygenic scores

Smith et al. (14) identified 12 T2D-associated genetic clusters based on GWAS summary statistics for 650 T2D-associated SNPs. These clusters reflect functional traits linked to T2D, including insulin deficiency (Beta Cell 1, Beta Cell 2, Proinsulin-negative); insulin resistance (Obesity, Lipodystrophy 1, Lipodystrophy 2, Hyper Insulin, Cholesterol-negative, Liver-Lipid, ALP-negative [Alkaline Phosphatase-negative]); mechanisms that are currently less well understood (Bilirubin, SHBG-LpA – characterized by reduced sex hormone-binding globulin and elevated lipoprotein(a)) (**Supplementary Table 1**).

pPGSs were calculated for each participant by multiplying the weight of each SNP by 0, 1, or 2 based on their genotype. Only 285 variants with weights > 0.7802 in any cluster were included, as recommended by Smith et al. (14) and consistent with prior studies (33, 34) to maximize the signal-to-noise ratio. These scores reflect an individual’s predisposition to specific T2D mechanisms.

### 2.8 SNP association analysis

For the 285 SNPs included in pPGSs calculations, associations with specific populations were assessed using regression tests from the PLINK package (ver. 2.00a5LM) (17), comparing one population against the rest.

### 2.9 pPGSs associations with populations, T2D, and clinical phenotypes

After generating individual-level pPGSs, we analyzed the association of the pPGSs with various clinical phenotypes, studied populations, and T2D, using linear regression (for continuous outcomes) or logistic regression (for binary outcomes). We tried to model T2D association with no covariates, adjusting for age, sex, and BMI as covariates; adjusting for the aforementioned covariates plus the inferred ancestries; or with these covariates plus the first 10 components of PCA.

## 3 Results

### 3.1 Patients with and without T2D fit the ancestry distribution

We analyzed the data of 275 eligible subjects, including 185 healthy donors and 90 patients with T2D. The median age of participants was 45 years, and 58.5% were women (**Figure 1A**). To validate self-reported ancestry, we first visualized the population structure using principal component analysis (PCA) with publicly available reference data (**Figure 1B**). The analysis revealed distinct clusters corresponding to different genetic ancestries, with most participants clustering according to their self-reported ancestry. To further verify global ancestry estimates, we used ADMIXTURE (**Figure 1C**). By analyzing all samples together, we identified nine stable ancestral components. Each population exhibited a unique ratio profile of these components. In this study, “ancestral components” refer to the distribution of specific genetic variants that vary across populations due to differences in ancestry. Based on these analyses, we categorized the study participants into three populations: Yakuts (62 healthy individuals and 30 T2D patients), Tatars (63 healthy individuals and 30 T2D patients), and Chechens (60 healthy individuals and 30 T2D patients).

### 3.2 Anthropometric and clinical metrics differ between ancestries

**Figure 2:**
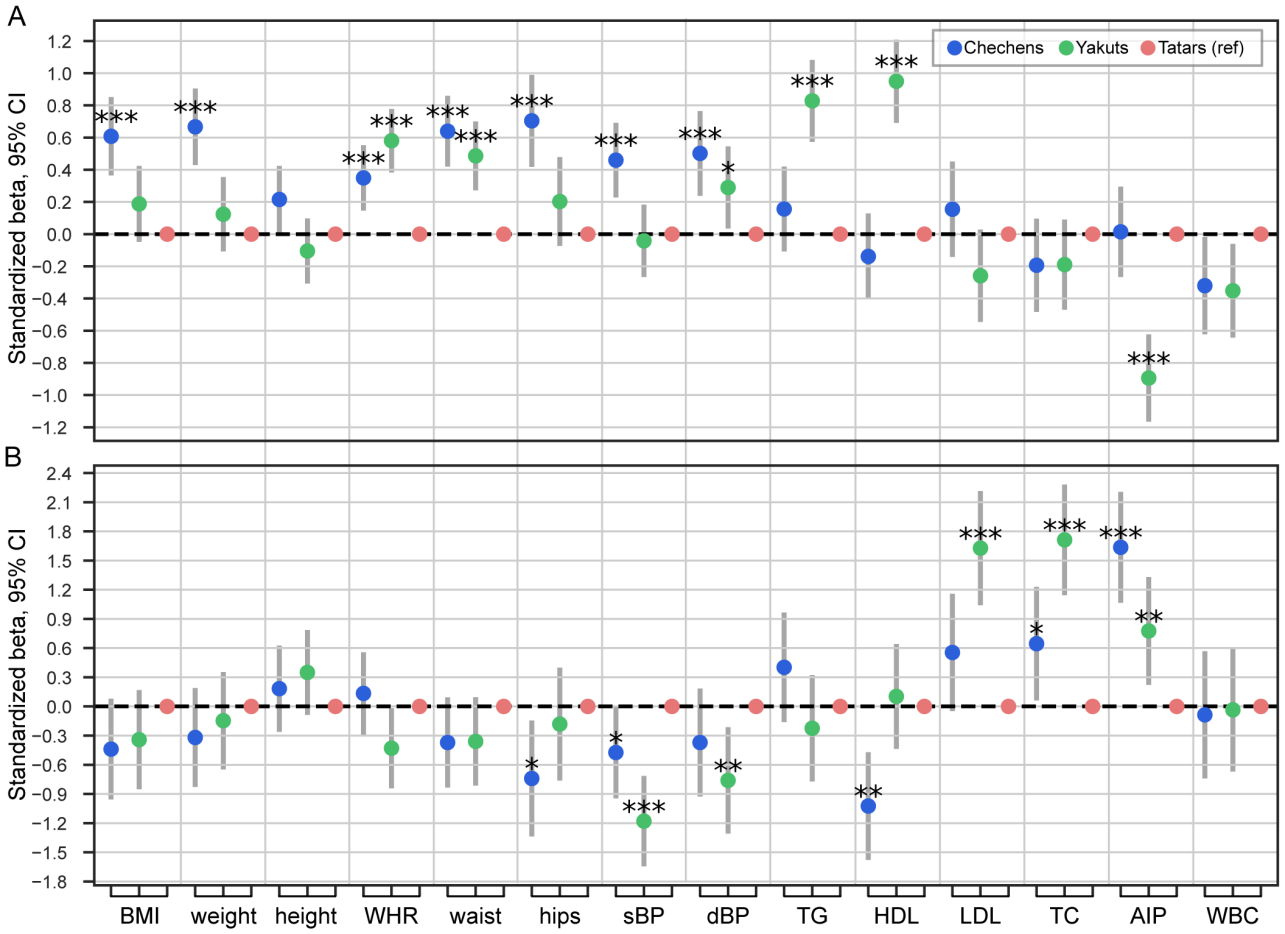
Association of ancestry and T2D (within an ancestry) with anthropometric and clinical measurements in individuals. **(A)** The estimated effect of ancestry on anthropometric and clinical measurements, adjusted for T2D status, age, and sex. **(B)** The estimated effect of T2D within a specific ancestry on anthropometric and clinical measurements, adjusted for ancestry, age, and sex. The Tatar population was used as a reference category. Adjusted p-value presented as *P≤ 0.05; **P ≤ 0.01; ***P ≤ 0.001. WHR, waist-hip ratio; sBP, systolic blood pressure; dBP, diastolic blood pressure; TG, triglycerides; TC, total cholesterol; AIP, atherogenic index of plasma; WBC, white blood cells count.

Descriptive statistics for anthropometric and biochemical measurements in the healthy control group and the T2D group are provided in **Supplementary Table 2** and **Supplementary Table 3**, respectively. To identify significant differences in the association of ancestry with anthropometric and clinical measurements, we performed regression analysis with adjustment for T2D status, age, and sex (**Figure 2A**). The Tatar population was selected as a reference category.

Our results indicate that the Chechen population exhibited the highest BMI (P<0.001), whereas Tatars and Yakuts had similar BMI levels. Waist-hip ratio (WHR) was the highest in Yakuts (P<0.001) followed by Chechens (P<0.001) and then Tatars. Waist circumference (WC) and diastolic blood pressure (dBP) were the highest in Chechens (P<0.001), followed by Yakuts (P<0.001) and then Tatars. Hips circumference (HC) and systolic blood pressure (sBP) were the highest in Chechens (P<0.001) and lower in Yakuts and Tatars. Looking at blood clinical characteristics, Yakuts differed from Tatars and Chechens by the highest triglycerides (TG) (P<0.001) and HDL levels (P<0.001). At the same time, Yakuts had the lowest atherogenic index of plasma (AIP) (P<0.001).

To investigate the effects that T2D had in a specific population, we performed the regression analysis of the interaction of ancestry and T2D features (‘population:T2D’) (**Figure 2B**), adjusting for the effects of ancestry, T2D status, age, and sex. Interestingly, most of the identified differences were associated with blood clinical parameters, not anthropometric measurements. Among patients with T2D, Chechen ancestry was associated with lower HC (P<0.05), sBP (P<0.05), and HDL levels (P<0.01) compared to Tatar ancestry. Belonging to Yakut ancestry was associated with the decrease in sBP (P<0.001), dBP (P<0.005), and the increase in LDL (P<0.005), TC (P<0.005), and AIP (P<0.005) compared to belonging to Tatar ancestry. Tatar ancestry itself within T2D was associated with the lowest TC and AIP levels, and the highest sBP among the populations.

### 3.3 T2D pPGSs demonstrate different ancestry distribution

**Figure 3:**
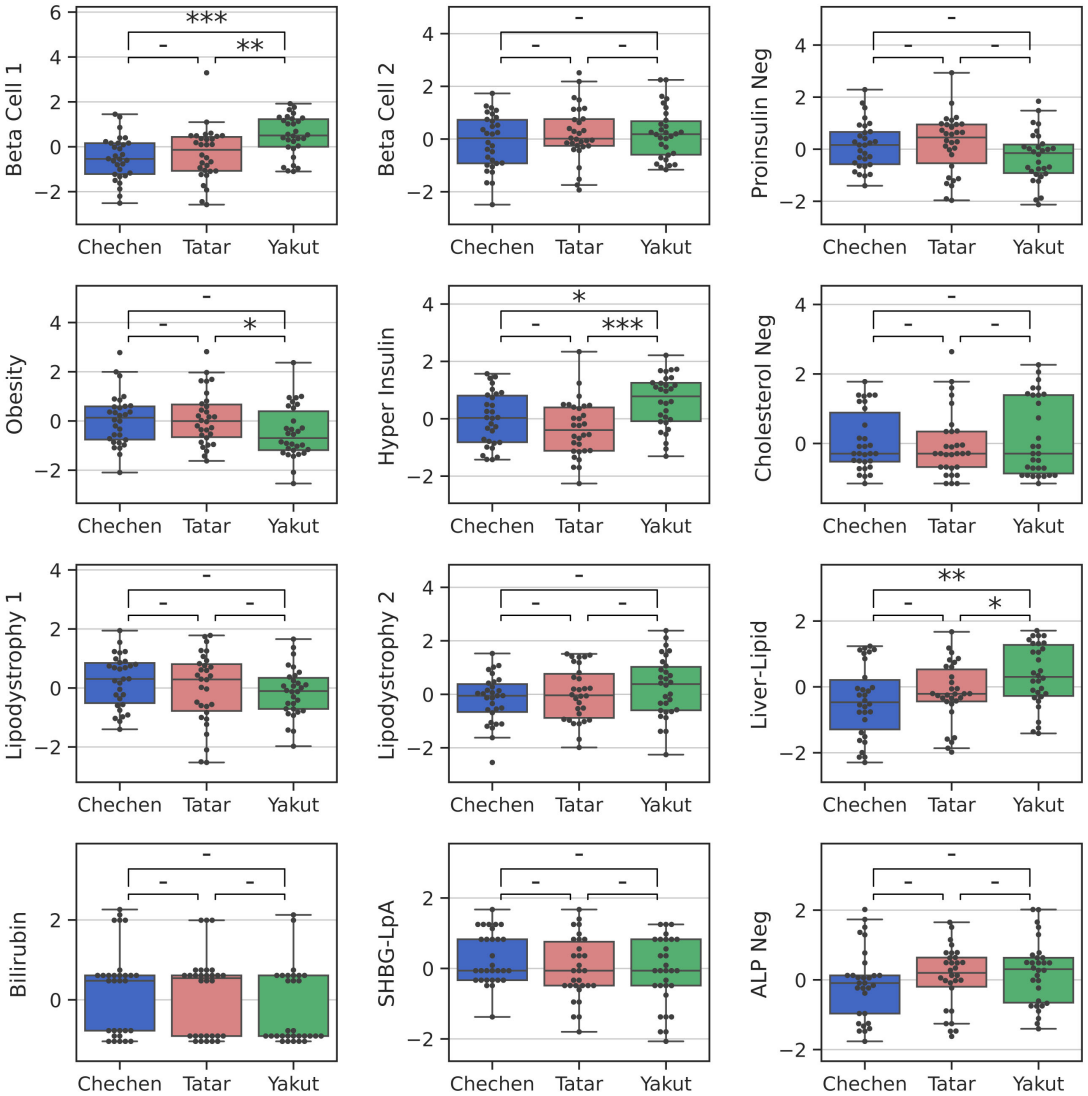
T2D genetic cluster pPGSs prevalence in the patients with T2D of different ancestries. P-values were assessed using the Mann-Whitney test, presented as *P≤ 0.05; **P ≤ 0.01; ***P ≤ 0.001.

To explore the genetic basis of ancestral differences and to investigate the genetic mechanisms underlying T2D, we next examined pPGSs distributions. We calculated the pPGSs of T2D genetic clusters proposed by Smith et al. (14) for each of the donors in our dataset. Among 285 SNPs included in all pPGSs, no SNPs demonstrated a significant frequency difference between any single population and the rest of the samples. However, several pPGSs exhibited ancestry-specific distributions among T2D patients (**Figure 3**). Specifically, Yakut T2D patients showed significantly higher pPGS for the Beta Cell 1, Hyper Insulin, and Liver-Lipid clusters compared to other populations (P ≤ 0.05, Mann-Whitney), while the Obesity pPGS (P ≤ 0.05) was lower in the Yakut T2D group.

We did not identify significant differences in the pPGS between healthy individuals and T2D patients, either across the entire cohort or within specific ancestry groups, except for ‘SHBG-LpA’ pPGSs, which showed a 4.64, 95% CI [1.56, 13.77] T2D odds ratio for Chechens (P ≤ 0.05). This outcome is not unexpected, as these pPGSs were not specifically designed to distinguish between individuals with T2D and those without the condition, and the sample size of the current study might be too small.

**Figure 4:**
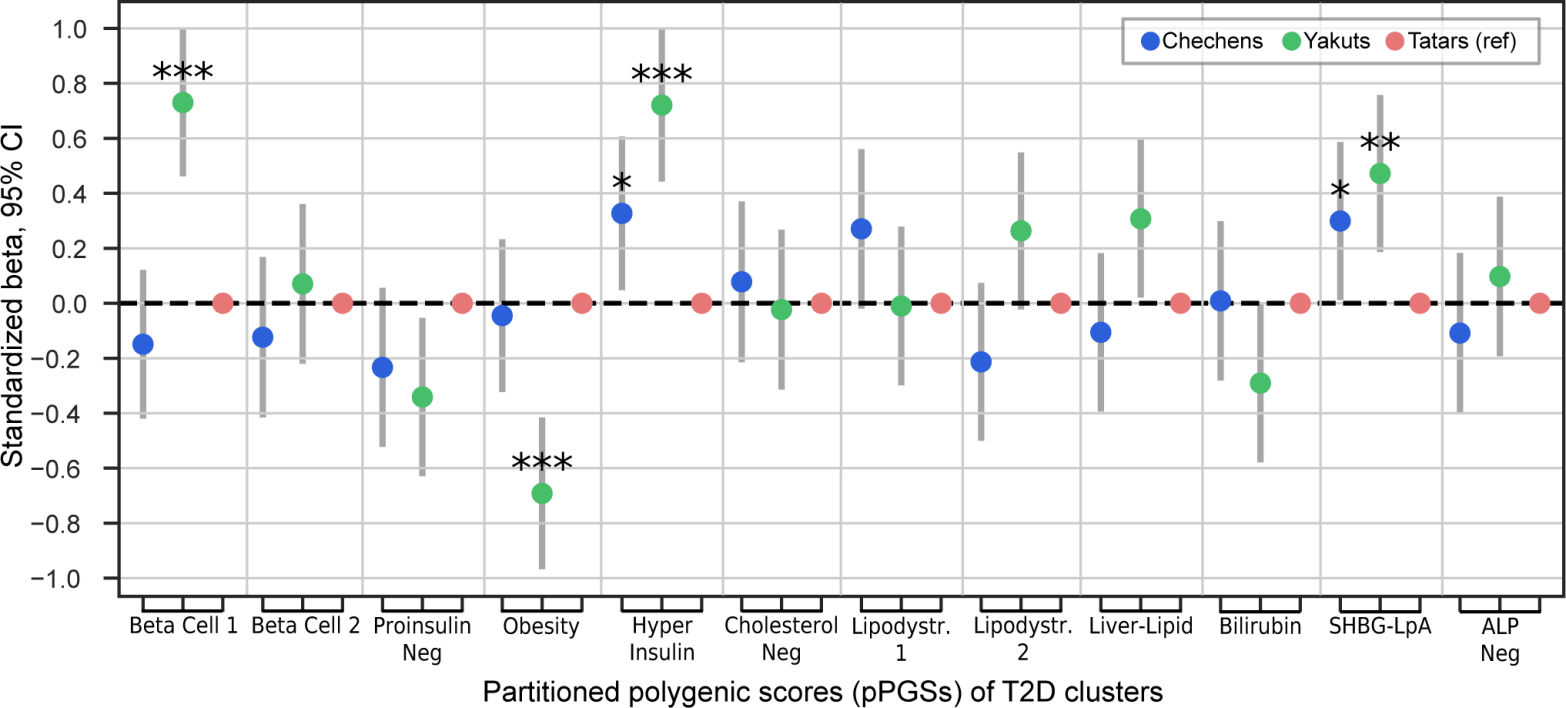
Cluster prevalence in the studied populations (individuals with T2D and non-T2D together). Linear regression was performed with adjustment for T2D status. Adjusted p-values (two-sided t-test) are presented as *P≤ 0.05; **P ≤ 0.01; ***P ≤ 0.001. CI, confidence interval.

To address the limitation of small sample size within each ancestry group, we evaluated pPGSs distributions across the entire cohort, adjusting for T2D status (**Figure 4**). The Tatar population was selected as the reference category. The Yakut population exhibited higher pPGS for the Beta Cell 1 (P ≤ 0.001), Hyper Insulin (P ≤ 0.001), and SHBG-LpA (P ≤ 0.01) clusters compared to Tatars. Conversely, Yakuts displayed the lowest Obesity pPGS (adj. P ≤ 0.001). Chechens showed higher Beta Cell 1 pPGS (P ≤ 0.05) than Tatars, but, lower than Yakuts. Tatars exhibited the lowest Hyper Insulin and SHBG-LpA pPGSs.

Interestingly, the Yakut population demonstrated the highest pPGS values for both the Beta Cell 1 and Hyper Insulin clusters, which are associated with contrasting phenotypic traits. The Hyper Insulin pPGS is linked to increased corrected insulin response (CIR) and disposition index (DI), whereas the Beta Cell 1 pPGS is associated with reduced CIR and DI. Analysis of the correlation between these pPGSs in the Yakut population and all populations combined revealed a weak association (Pearson’s correlation = 0.07) (**Supplementary Figure S2**).

### 3.4 T2D genetic clusters show variation in the ancestry space

**Figure 5:**
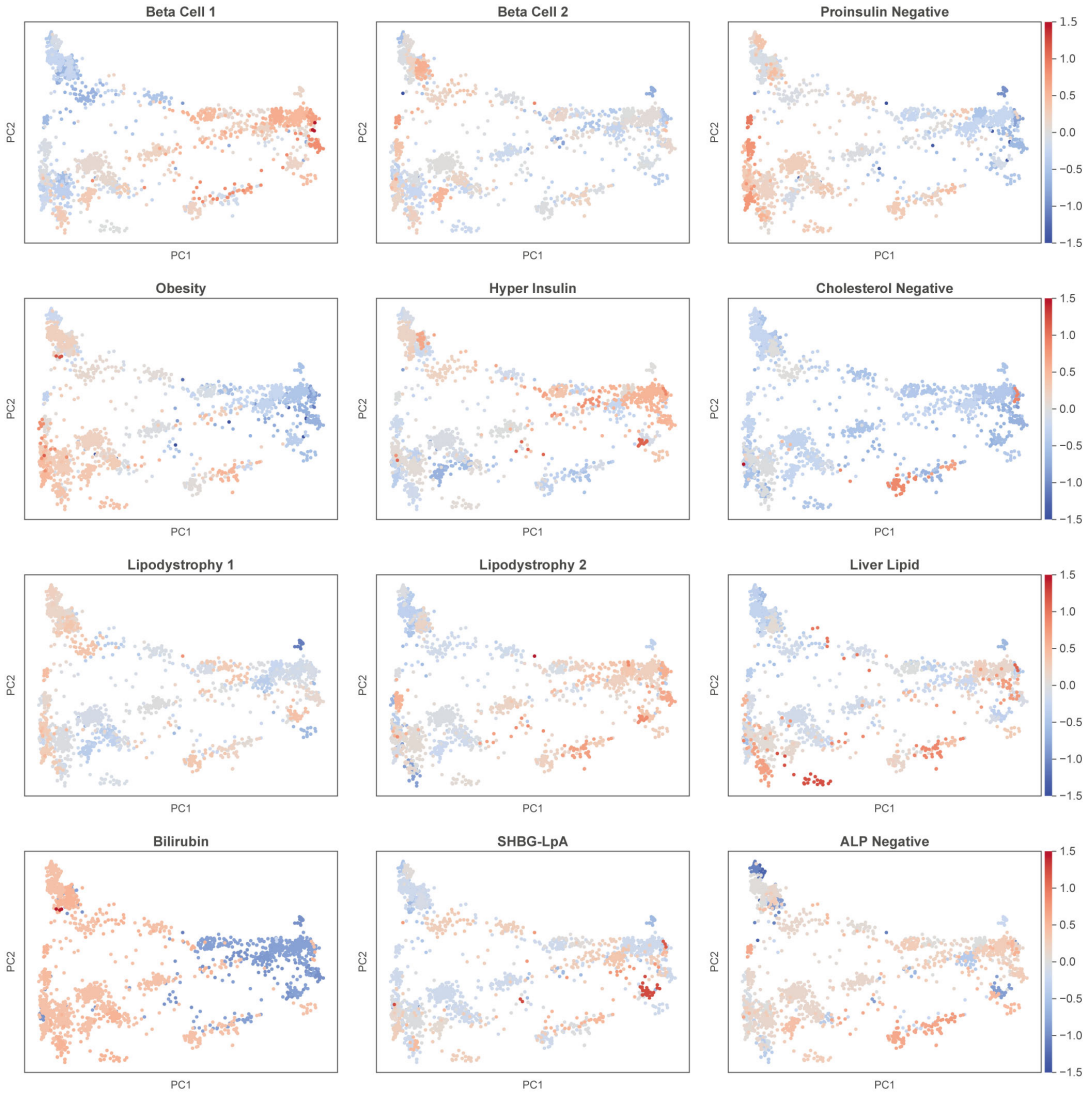
pPGSs distribution in the combined data of the study and public ancestry reference. The same PCA components as in **Figure 1B** are used. Population clusters are colored based on the median value of a specific pPGS.

To explore the variation in T2D mechanistic pathways across different genetic ancestries, we analyzed pPGSs for T2D clusters using a combined dataset that included both our study participants and publicly available ancestry-annotated data (**Figure 5**). This approach allowed us to assess the distribution of pPGSs across a broader genetic ancestry spectrum. Although T2D status information was unavailable for these samples, the results remained consistent. pPGSs for Beta Cell 1, Hyper Insulin are dramatically higher in eastern populations, while pPGS for Obesity is higher in western populations. We found that the distribution reflected known population characteristics from the literature. For instance, the Khanty population showed one of the highest scores for the Cholesterol Negative pPGS, which aligns with previous studies reporting their significantly lower cholesterol levels compared to Europeans (35). The results reveal distinct patterns of pPGS variation, underscoring the influence of genetic ancestry on T2D-related pathways.

## 4 Discussion

Our analysis of anthropometric and clinical data across the three studied populations — Chechens, Tatars, and Yakuts — revealed significant ancestry-specific differences in phenotypic traits and T2D-associated genetic clusters. Chechens exhibited higher BMI and weight compared to Tatars and Yakuts, consistent with findings by Kononenko et al. (12), who reported that North Caucasus populations, including Chechens, have higher BMI but a lower prevalence of carbohydrate metabolism disorders (e.g., T2D and prediabetes) compared to other Russian populations. This discrepancy may be attributed to the presence of genetic protective traits in Chechens. The findings align with the study by Loos&Yeo (36), which suggests that genetic factors can modulate metabolic disease risk even in the presence of obesity.

In contrast, the Yakuts, the closest population to the East-Asian populations (28, 37), displayed distinct metabolic profiles, including the highest waist-hip ratio (WHR), triglycerides (TG), and HDL levels, alongside the lowest atherogenic index. These observations are in line with the traits of the East-Asian populations and may reflect adaptations to chronic cold exposure, as observed in Arctic populations, where improved fat utilization, increased insulin sensitivity, and reduced circulating insulin concentrations have been documented (38, 39). The Yakut population’s pPGS profile, characterized by elevated Liver-Lipid (especially in T2D patients) and reduced Obesity scores, supports this hypothesis. Yakuts showed higher pPGS for Beta Cell 1, suggesting that β-cell dysfunction pathways play a larger role in T2D pathogenesis for them than obesity-related insulin resistance. The same fact was shown by Yabe et al. for East Asians in comparison to Caucasians (8). Increased Hyper Insulin pPGS in Yakuts suggests compensatory insulin production, often seen in early disease stages. Interestingly, Yakuts exhibited the highest pPGS for the phenotypically opposing Beta Cell 1 and Hyper Insulin clusters. However, the lack of correlation between these scores suggests that these traits are driven by distinct genetic mechanisms within the population.

Tatars, on the other hand, showed the lowest WHR, waist circumference and diastolic blood pressure (dBP) levels. These findings highlight the unique characteristics of Tatars, which may contribute to their distinct T2D risk profile. The decreased Hyper Insulin and SHBG-LpA pPGSs in Tatars further underscore the role of ancestry-specific genetic factors in shaping T2D pathophysiology.

Our results underscore the importance of studying diverse ancestral populations to uncover population-specific genetic and metabolic traits. However, several limitations must be acknowledged. First, the relatively small sample sizes within each ancestry group may have limited our ability to detect significant associations, particularly for rare variants. This issue is compounded by sampling bias, which can affect allele frequency estimates and ancestry inference, as discussed by Risso et al. (40), Shringarpure & Xing (41), and Marchini et al. (4). Second, the availability of genetic data from the Russian Federation remains limited, with fewer than 2,000 publicly available genomes, predominantly of European ancestry (**Supplementary Figure S1**). This highlights the need for broader genetic research initiatives to include underrepresented populations, as emphasized in large-scale multi-ancestry studies (42).

Discovery and transferability studies across ancestries promise to be integral in advancing our understanding of the genetic basis of T2D and providing insights into differences in the prevalence and physiology of the disease between ancestries. Our results emphasize the importance of analyzing diverse ancestral populations. Translating genetic discoveries into clinical practice remains challenging, mainly due to the abundance of non-coding variants and the complex interplay of multiple genetic and environmental factors (4). However, innovative approaches, such as pPGSs, offer promising avenues for understanding T2D heterogeneity. pPGSs, which cluster genetic loci based on shared association patterns, provide insights into distinct pathological pathways and can guide personalized treatment strategies. For example, individuals with a high pPGS for β cell dysfunction may benefit from early interventions to preserve β cell function. In contrast, those with a high pPGS associated with insulin resistance mechanisms may require targeted therapies to improve insulin sensitivity. Summing up, studying these T2D-related biological processes could help move closer to personalized treatment plans for patients, based on their genetic makeup.

The low representation of ancestral diversity in modern population studies reduces our ability to translate genetic research into clinical practice, making conclusions dangerously incomplete or even flawed. For example, attempts to apply genetic risk estimates obtained from Western Europeans to Russian populations may lead to underestimating the risk due to differences in allele frequencies and genetic architecture. This underscores the need for large-scale genetic studies in Russia to identify population-specific risk variants and improve the accuracy of risk prediction models.

Despite these limitations, our findings contribute to a growing body of evidence demonstrating the utility of pPGS in elucidating T2D mechanisms. Building on Smith et al.’s framework of T2D clusters (14), our work uniquely applies this method to underrepresented populations in Russia. While pPGS provide valuable insights into the genetic architecture of T2D, their clinical application requires further refinement. For instance, current pPGS models cannot definitively assign individuals to specific genetic subtypes, and functional validation through experimental models is needed to confirm the biological relevance of identified clusters (34). Nevertheless, the use of pPGS as mechanistic “signatures” holds promise for improving T2D stratification and advancing precision medicine approaches.

In conclusion, our study demonstrates that similar patterns of T2D genetic clusters occur across multiple populations but with varying frequencies. These ancestry-specific differences in genetic and metabolic traits underscore the importance of considering genetic background in T2D risk assessment and management. Future research should focus on expanding genetic datasets to include underrepresented populations, validating pPGS in experimental models, and developing precision medicine strategies tailored to specific T2D mechanisms. Such efforts will deepen our understanding of T2D heterogeneity and pave the way for more personalized and effective interventions.

## Data Availability

The original contributions presented in the study are included in the article/**Supplementary Material**. Further inquiries can be directed to the corresponding author.
