# Supplementary material for: Genetic and phenotypic heterogeneity of type 2 diabetes across Russian ancestry groups
Source: Front Endocrinol (Lausanne). 2025 Sep 10;16:1672403. doi: 10.3389/fendo.2025.1672403 (PMC12457129; doi:10.3389/fendo.2025.1672403)
Supplement: Supplementary file 1 [file DataSheet1.pdf]

Supplementary Material

1 Supplementary Methods

1.1 Determination of genetic ancestry

Samples with ancestry admixture profiles significantly deviating (Pearson's cor. < 0.7) from their reported population averages were excluded. These samples included:

- 4461875862_R02C02 annotated as Tatar from Triska et al. (1) (due to a >89% Caucasian component, which was atypical for Tatars)
- HGDP00959 annotated as Yakut from HGDP (2) (due to an unusually high East-Asian component)

1.2 References

1. Triska P, Chekanov N, Stepanov V, Khusnutdinova EK, Kumar GPA, Akhmetova V, et al. Between Lake Baikal and the Baltic Sea: genomic history of the gateway to Europe. BMC Genet. 2017 Dec 28;18(1):110.

2. Bergström A, McCarthy SA, Hui R, Almarri MA, Ayub Q, Danecek P, et al. Insights into human genetic variation and population history from 929 diverse genomes. Science. 2020 Mar 20;367(6484):eaay5012.

2 Supplementary Figures

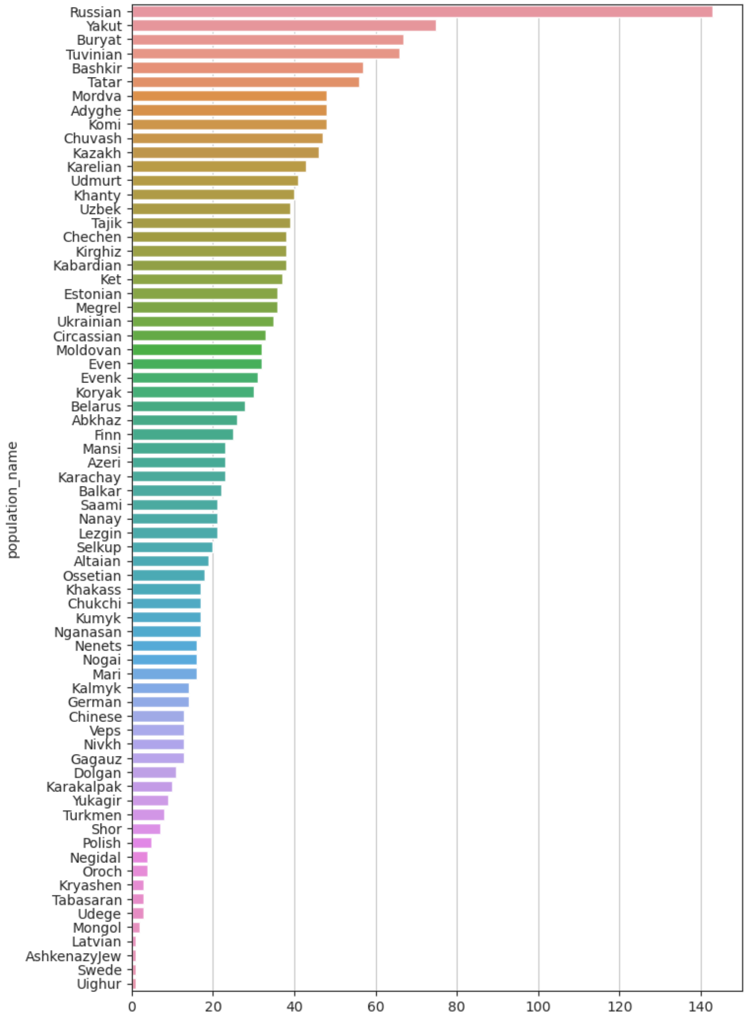

Supplementary Figure 1. The populations present in the ancestry reference data. The data was collected from publicly available sources.

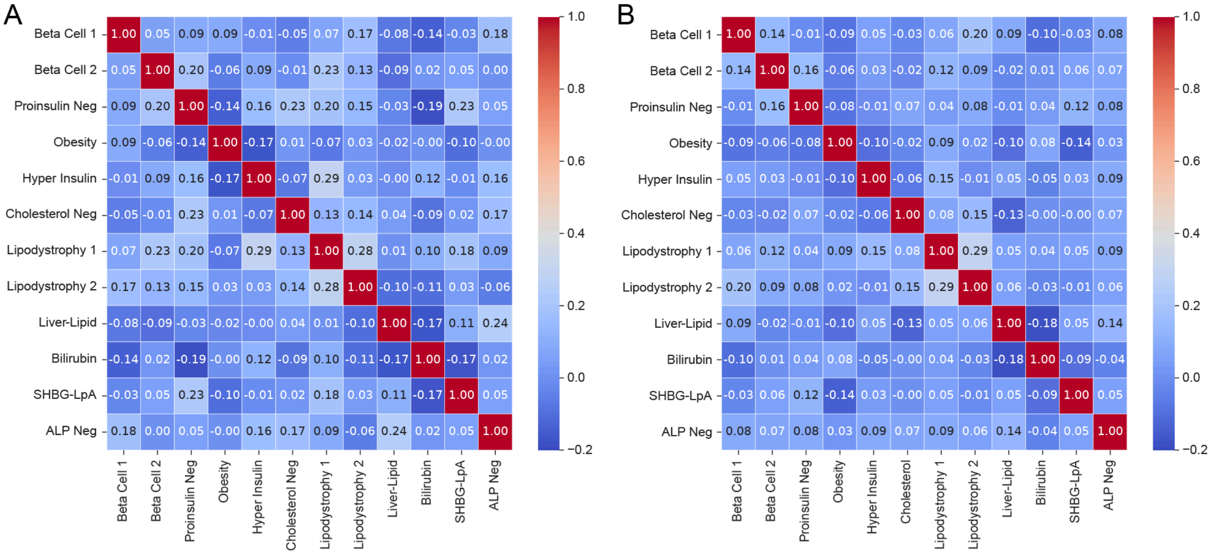

Supplementary Figure 2. Correlation of pPGSs. Analyzed data consists of the data from the current study of individuals belonging to the studied ancestries. (A) Pearson correlation of pPGSs of individuals belonging to the Yakut ancestry. (B)Pearson correlation of pPGSs of individuals belonging to the Chechen, Tatar and Yakut ancestries.
